# Supplementary figures and images for: Low-Cost Chlorophyll Fluorescence Imaging for Stress Detection
Source: Sensors (Basel). 2021 Mar 15;21(6):2055. doi: 10.3390/s21062055 (PMC7999708; doi:10.3390/s21062055)

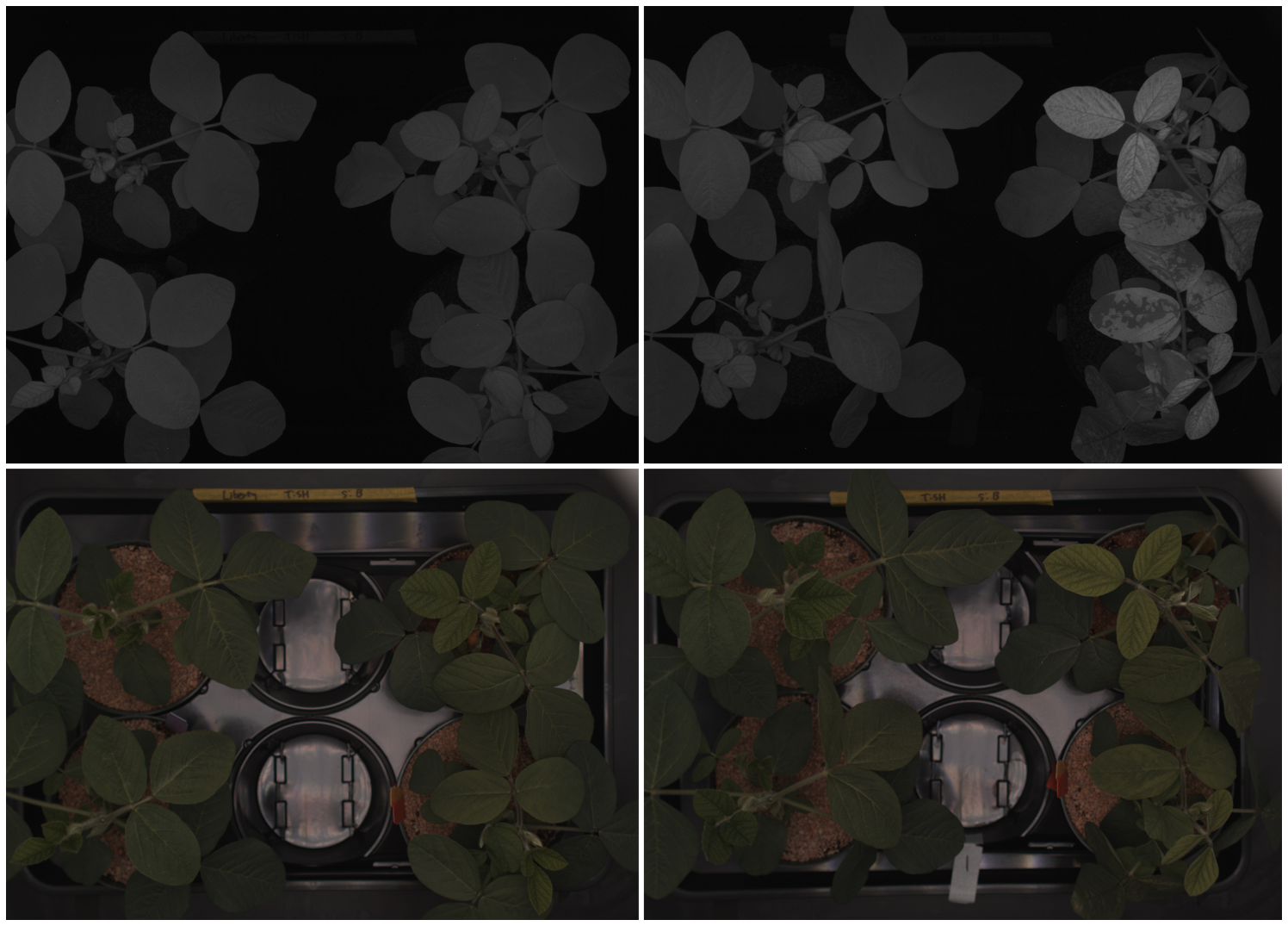

Supplement: Supplementary file 1 [file sensors-21-02055-s001.zip › Supplemental Figure 5.png]

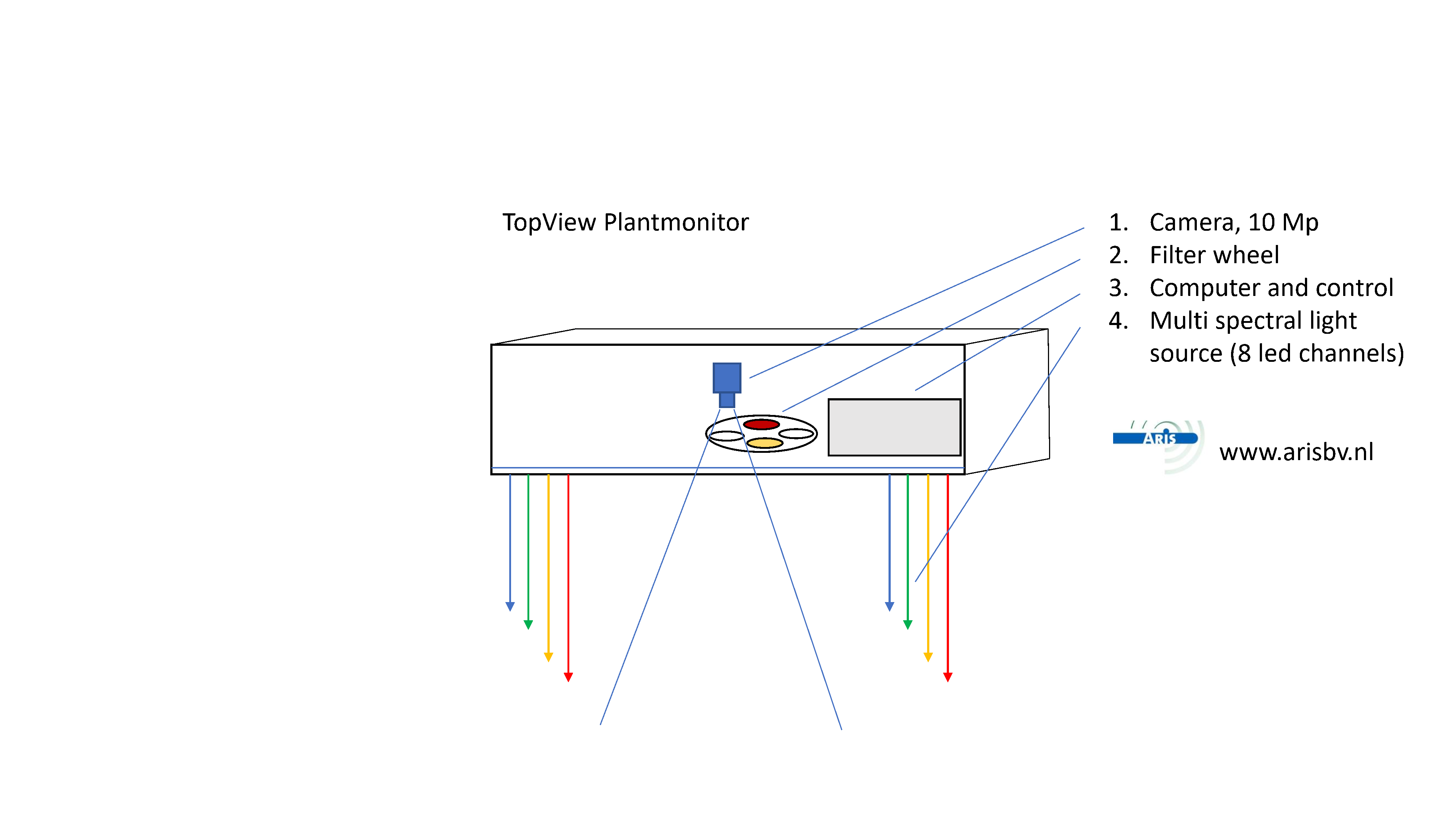

Supplement: Supplementary file 1 [file sensors-21-02055-s001.zip › Supplemental Figure 1.tiff]

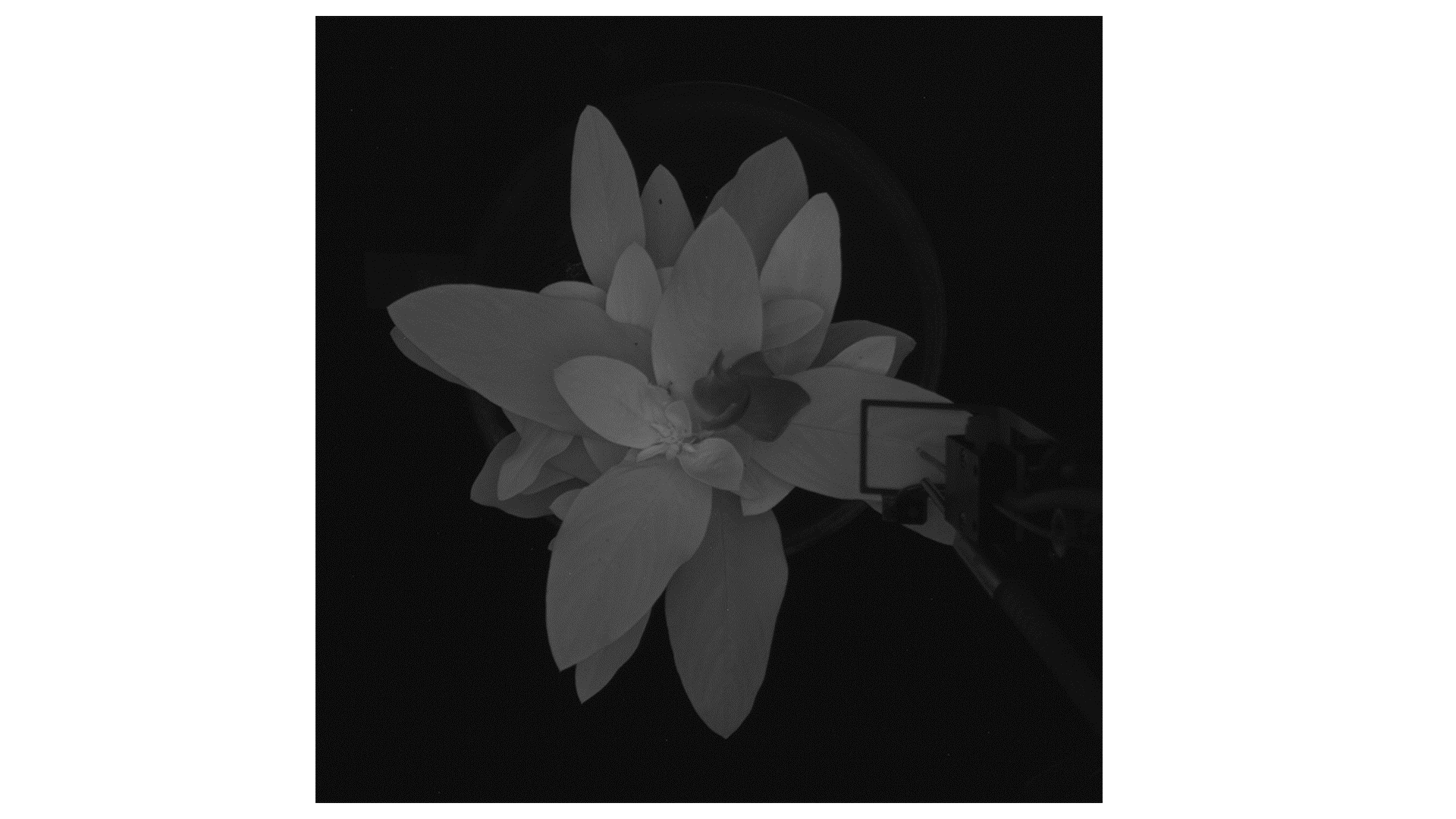

Supplement: Supplementary file 1 [file sensors-21-02055-s001.zip › Supplemental Figure 2.gif]

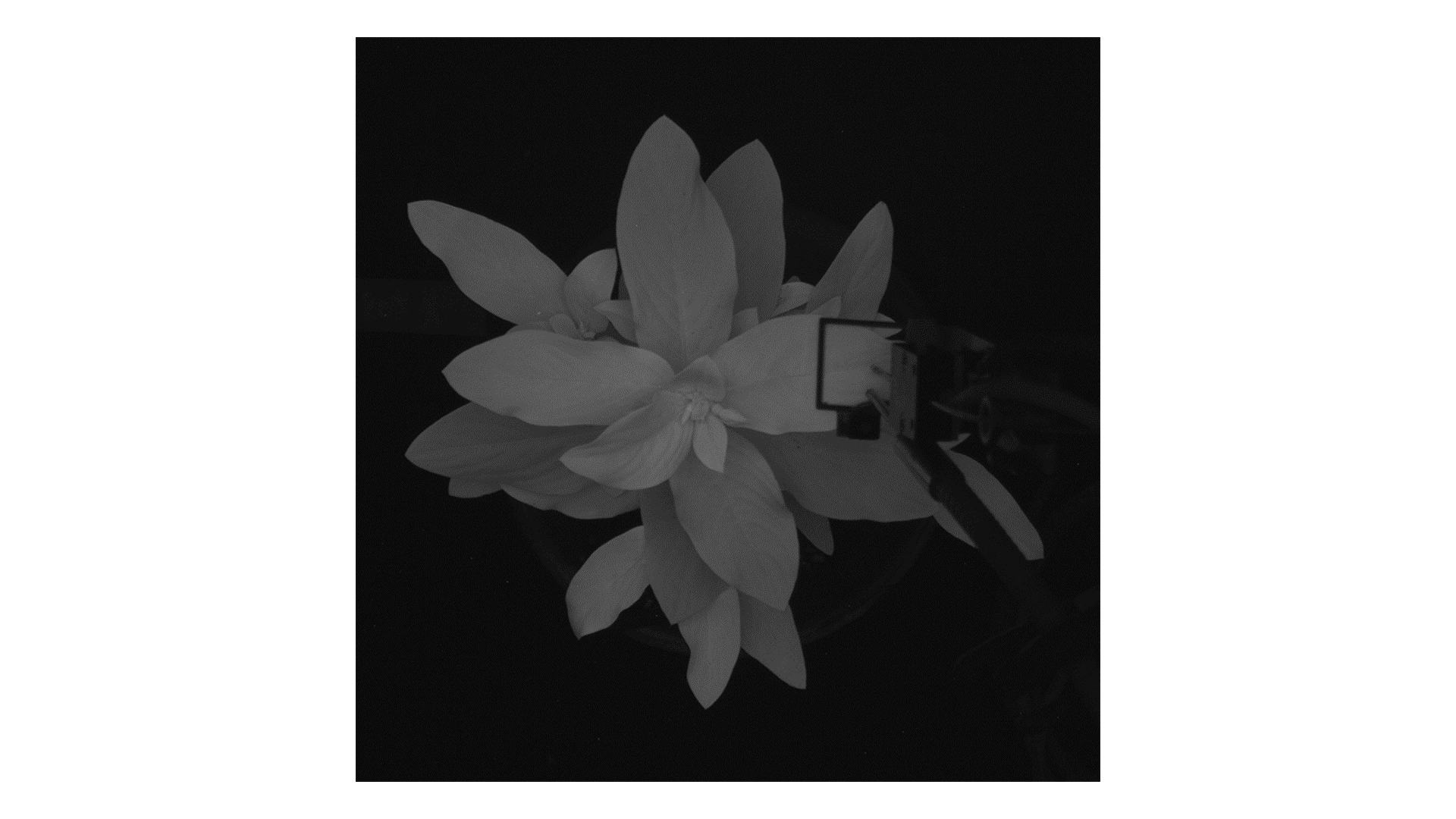

Supplement: Supplementary file 1 [file sensors-21-02055-s001.zip › Supplemental Figure 3.gif]

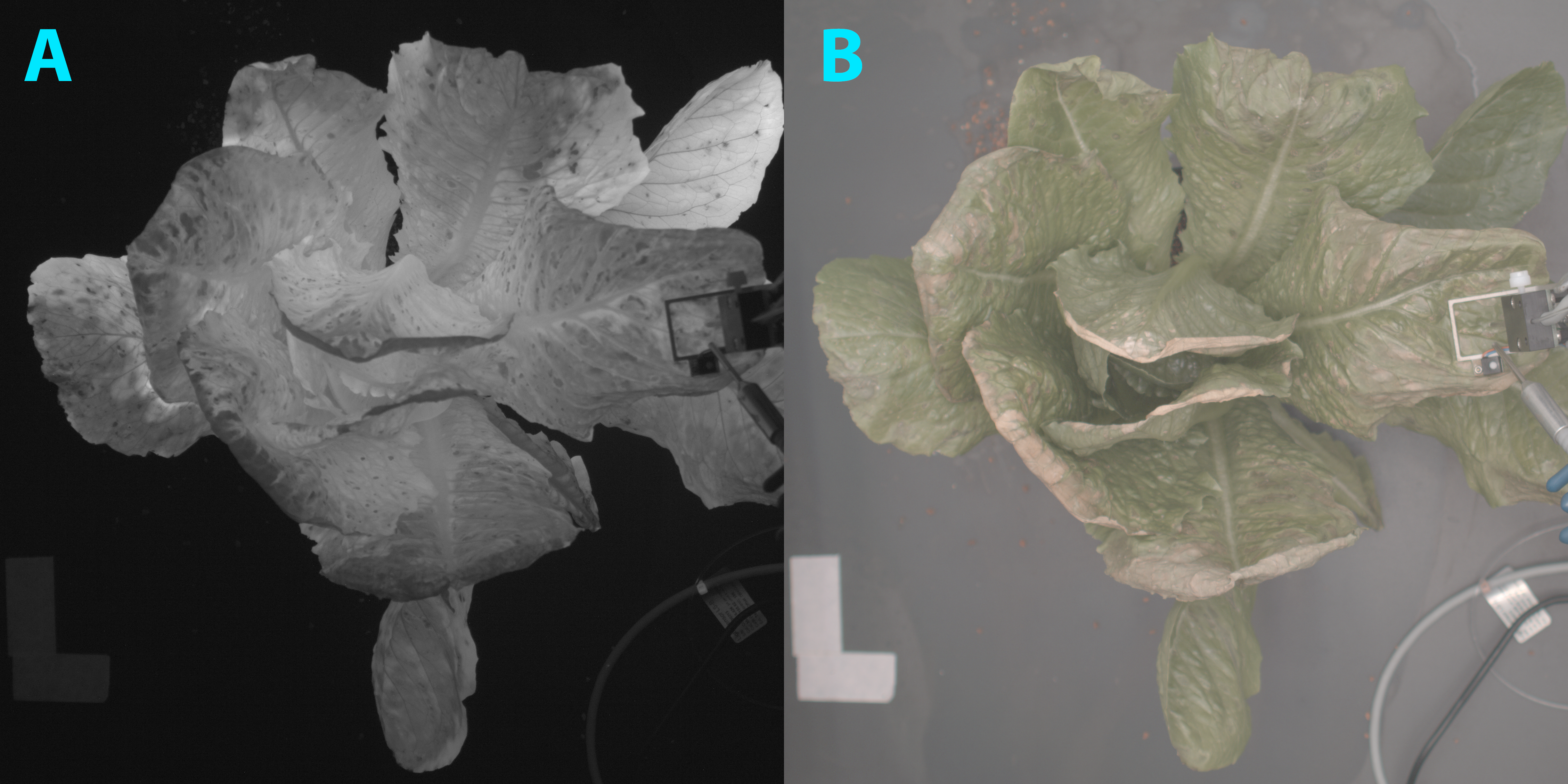

Supplement: Supplementary file 1 [file sensors-21-02055-s001.zip › Supplemental Figure 4.png]
